# Supplementary material for: Are carbon emissions trading and green financial instruments synergistic? -Comprehensive quantitative research based on content analysis
Source: PLoS One. 2024 Mar 7;19(3):e0298601. doi: 10.1371/journal.pone.0298601 (PMC10919601; doi:10.1371/journal.pone.0298601)
Supplement: S1 Data — (ZIP) [file pone.0298601.s001.zip › Data/地级市Z_F工作报告.zip!/╣π╬≈/║╪╓▌╩╨/└┤╫╘╣1⁄2╓┌║┼í╛┬φ┐╦╩2╛▌═°í┐_2008.docx]

各位代表：

现在，我代表市人民政府向大会报告工作，请予审议，并请市政协各位委员和其他列席会议的同志提出意见。

一、2007年工作回顾

2007年是我市经济社会发展取得显著成绩的一年。一年来，在市委正确领导下，在市人大及其常委会和市政协监督、支持下，新一届市人民政府和全市各族人民一道，全面落实科学发展观，加快推进“三化两业”，克服发展中遇到的各种困难和问题，较好地完成了市二届人大二次会议确定的各项目标任务，实现了全市经济社会又好又快发展。

——经济保持强劲增长，经济总量不断扩大。初步统计，全市实现生产总值232.76亿元,增长14.5 %,比年初计划增2.5个百分点；一、二、三次产业分别增长5.5%、19.4%和15.8 %。全市财政收入15.81亿元,增长16.6%，比年初计划增1.6个百分点;全社会固定资产投资总额133.66亿元,增长26.1 %，比年初计划增6.1个百分点；全部工业总产值266.39亿元, 增长29.1 %，比年初计划增5.92个百分点，其中规模以上工业总产值115.09亿元，增长48.3%，比年初计划增17.89个百分点；农业总产值108.92亿元, 增长5.5%，比年初计划增0.5个百分点；社会消费品零售总额47.11亿元，增长19.2%，比年初计划增7.2个百分点；外贸出口总额8614万美元，完成年初计划目标；城镇居民人均可支配收入10790元，增长25.2%，比年初计划增15.2个百分点；农民人均纯收入3146元，增长17.3%，比年初计划增5.8个百分点。

——工业经济提速发展，工业化步伐加快。“工业立市”战略深入实施，工业经济规模和效益创历史新高，全市实现工业增加值94.15亿元，完成年初计划的104.61%，增长21.1%，工业增加值占GDP比重达40.4%，对经济增长的贡献率达55.1%。工业结构不断优化，电子、有色金属开采加工、林产、建材、电力、食品药品加工等工业支柱进一步壮大。工业项目建设快速推进，红星纸业特种纸、嘉和木业中纤板、金源稀土钕铁硼永磁材料等25个重点工业项目投产，新增工业产值22亿元。工业技改取得新突破，全年实施工业技改项目175项，完成技改投资14.86亿元，增长26%。工业园区建设取得新成效，入园企业不断增多，旺高、信都、西湾（平桂）工业区新引进项目42个，总投资14.12亿元，完成投资8.1亿元。

——城市规划建设进一步完善，城乡面貌显著改观。城市规划体系不断完善，修编调整贺州市城市规划控制区，完成了贺州市城市近期建设规划、住房建设规划、城市消防规划等一批专项规划和旧飞机坪详规、火车站片区详规等31个详细规划。成立了平桂管理区，调整完善了行政区划。市政项目建设进展顺利，垃圾处理厂一期工程、灵峰广场商业步行街、鼎富装饰广场、阳光财富等一批项目投入使用；汇豪国际城、远东国际城、职教中心、体育中心等项目加快建设，污水处理厂项目正式开工。城市经营和管理水平不断提高，“城乡清洁工程”深入实施，市容市貌进一步改善，城市品位明显提升，“五年创新城”目标基本实现，获全区第六届城市市容环境综合整治南珠杯竞赛城市B类特等奖，昭平县、钟山县分获县城A类特等奖、优秀奖，富川县获县城C类特等奖，八步区信都镇获乡镇特等奖。

——农业企业化成效显著，新农村建设扎实推进。在稳定粮食生产基础上，特色农业、品牌农业、生态农业得到巩固和发展，优势农产品基地进一步扩大。生态林业稳步发展，全市完成速丰林造林22.02万亩，新建沼气池14567座。农产品标准化生产全面推广，农业企业化经营取得新成效。全年新增规模农业企业35家、新增农民专业合作社8家；全市落实订单农业面积45万亩，新增带动农户3万多户。农业招商引资力度加大，成功举办第四届农产品交易暨农业项目洽谈会、昭平茶王节、富川脐橙节。选派1474名干部驻村担任社会主义新农村建设指导员，扎实推进新农村建设。农田水利建设、病险水库除险加固和农村人饮安全解困工作取得新成绩，群众生产生活条件不断改善，农民收入明显增加。

——商贸旅游业日益繁荣，第三产业快速发展。成功实现创建“中国优秀旅游城市”目标。各主要旅游景区景点的基础设施建设不断加强，城市旅游功能进一步完善；旅游宣传促销成效明显，古镇文化、客家文化特色旅游加快发展，旅游知名度进一步提高；旅游行业规范管理取得新成效，旅游环境不断优化。全年接待国内外游客327.2万人次，增长5.71 %，其中境外游客12.2万人次，增长12.5%;旅游总收入18.17亿元,增长16.2 %。商贸流通基础设施建设继续加强，一批专业市场加快建设，部分市场项目竣工使用，“万村千乡” 市场工程深入实施，完成100家农家店建设改造任务。房地产、餐饮、中介、金融、保险等其他服务业也快速发展。

——招商引资实现新突破,迎接产业转移取得新成效。组团参加第四届中国——东盟博览会、第四届中国国际中小企业博览会暨中日中小企业博览会，在澳大利亚及深圳、泉州举办投资推介会，组织举办贺州石材重钙产品展销暨项目招商洽谈会、贺州桂台客属联谊会等重大招商推介活动，入选“中国最佳粤商投资城市”。加强招商引资，选派120名领导干部赴广州、佛山、温州等地学习培训、挂职招商；制定优势企业“以商招商”工作方案，推出80个以商招商项目。加快迎接东部产业转移基地建设，认真落实“百企入桂”各项措施，推动一批产业转移项目落户贺州。全年新引进内资项目689个，合同投资额185.7亿元，实施项目到位资金102.9亿元，增长45.3%；引进外资项目14个，实际利用外资2336万美元，增长79.1%。

——项目建设加快推进，固定资产投资再创新高。百项工程和交通基础设施建设大会战取得实效，事关贺州发展大局的桂梧高速公路、洛湛铁路、广贺高速公路贺州段加快建设，永贺高速公路、贵广快速铁路项目前期工作顺利开展，总装机500万千瓦的贺州火电厂一期装机200万千瓦工程、年产400万吨水泥的贺州华润水泥厂第1条200万吨生产线、林浆纸板一体化等项目开工准备工作加紧推进。年内列入自治区层面统筹推进的4个新开工重大项目有3个项目实现开工；市级层面统筹推进的23个重点项目开工建设18个。全年新开工投资规模50万元以上项目724个，项目总投资100.2亿元,增长56%,完成投资48.9亿元,增长74 %。

——社会事业和谐发展，人民生活显著改善。“民心工程”深入实施并不断拓展。五保村长效管理逐步规范。新型农村合作医疗扎实推进，农民参合率达74.26%。社会救助体系不断完善，养老、失业、医疗、工伤、生育保险覆盖面进一步扩大。就业和再就业工作稳步推进，全年新增城镇就业1.28万人，完成计划任务的128%，城镇登记失业率为3.56%。民政救灾救济工作成效显著，农村低保工作全面启动，城镇低保进一步规范。农村特困户危房改造工程、优抚对象危房改造工程等工作有序推进。整村推进贫困村扶贫开发取得实效，解决1.7万人农村绝对贫困人口的温饱问题，完成计划任务的200%；农村低收入贫困人口脱贫1.58万人，完成计划任务的158%。农村计划生育奖励扶助制度进一步完善和落实，低生育水平持续稳定，荣获“全国婚育新风进万家活动示范市”称号。科技创新成效明显，第三轮科技创新计划顺利通过自治区检查验收。教育事业加快发展，“两基”达标工作顺利通过国家检查验收。人才队伍建设不断加强。新闻出版业持续健康发展，版权保护和“扫黄打非”工作取得新成效。广播电视“村村通”、电影进村、送戏下乡等活动积极推进。文化事业健康发展，群众性文化活动广泛开展。 “关爱功臣”活动全面开展。精神文明建设卓有成效，荣获“全国双拥模范城”称号。“平安贺州”和“三无村屯（社区）”创建工作深入开展，安全生产、信访和人民调解工作进一步加强，治安形势继续好转。环保专项行动扎实开展，节能减排取得成效，全市单位生产总值能耗降低2.5%，超额完成年初计划降2.1%的目标；主要污染物排放总量减少8.3%，超额完成年初计划减3.2%的目标，贺江等主要河流水质得到改善，解决了一批影响人民群众生产生活的环境问题。国防动员、民兵预备役等工作继续加强，体育、妇女儿童、老龄、残疾人事业以及人事、机构编制、工商管理、食品药品监督、技术监督、出入境检验检疫、审计、统计、物价、民族、宗教、外事、侨务、对台、防震减灾、应急、气象、农机、修志、档案、工会、住房公积金等工作取得新进步。

——政府自身建设进一步加强，机关行政效能明显提高。在全区率先建立市政府采购招投标中心，建设工程招投标、经营性土地出让、政府采购、产权交易等领域的制度建设进一步规范。严格依法行政，加强电子政务建设，推进政务公开，充分发挥市长热线、市长电子信箱、行风热线等信息互动平台的作用，政府工作透明度不断提高。转变干部作风加强机关行政效能建设活动深入开展，首问负责制、限时办结制、责任追究制“三项制度”全面落实。完善各级政务中心建设，认真清理行政审批项目，全面推行行政审批“一站式”服务，政务环境不断优化，机关行政效能进一步提高。加强廉政建设，加大对商业贿赂和损害群众利益不正之风的治理力度，严肃查处了一批违法违纪案件。自觉接受人大依法监督和政协民主监督，认真办理人大代表建议和政协委员提案。推行村务公开，强化社区管理，加强普法教育，开展法律援助，基层民主和法制建设不断深入。开展产品质量和食品安全专项整治，市场环境不断优化。完善应急预案，健全管理网络，突发公共事件应急管理水平得到提高。开展信访下访活动，坚持领导干部开门接访、带案下访，一批群众关注的热点难点问题得到解决。

各位代表，过去的一年，我们面临宏观环境趋紧、土地、资金制约、居民消费品价格持续走高、煤电油运紧张等诸多困难，取得以上成绩,的确来之不易。这是上级党委、政府和市委正确领导的结果，是市人大、政协监督支持的结果，是全市人民团结拼搏、克难攻坚的结果。尤其要指出的是，今年1月中旬至2月上旬，我市出现了严重的低温雨雪冰冻灾害，时间之长、强度之大、灾情之重，历史罕见。面对突如其来的严重冰冻灾害，在党中央、国务院的亲切关怀下，在自治区党委、自治区人民政府和市委的正确领导下，经过全市人民半个多月的顽强奋战，没有因灾冻死、饿死一个人，确保了社会秩序井然、市场供应充足、群众生活安定、经济运行平稳，把灾害损失降低到了最低程度，取得了抗冰救灾工作的阶段性胜利。在这场抗冰救灾斗争中，全市各级领导干部分片负责，深入一线指导抗冰救灾，广大党员干部身先士卒、冲锋在前，表现出崇高的政治觉悟和良好的精神风貌；驻贺部队、武警官兵、民兵预备役官兵、公安干警充分发扬不畏艰险、英勇拼博的抗冰救灾精神；全市广大人民群众不等不靠、不退缩、不观望，以各种方式顽强地同坚冰搏斗，在积极开展自救的同时，努力为社会贡献自己的爱心，汇聚出“万众一心、共抗冰灾”的强大力量。这股强大的力量，也是激励我们建设富裕和谐新贺州的宝贵的精神财富。在此，我代表市人民政府，向全市人民，向驻贺部队、武警官兵和公安干警，向各民主党派、工商联、无党派人士、人民团体以及所有关心、支持贺州建设和发展的各界人士，表示衷心的感谢和崇高的敬意！

在肯定成绩的同时，我们也清醒地看到，我市在发展中还存在不少困难和问题，主要是：经济总量小，产业结构不够合理，财力仍然薄弱；制约经济发展的结构性矛盾依然突出，经济运行的质量和效益有待提高；节能减排压力较大；科技创新能力不强；基础设施短期内尚未得到根本改善；城镇化水平还不高；农民增收难度加大；就业形势依然严峻；一些工作如各县城污水处理厂和垃圾处理厂建设尚未启动；干部队伍思想还不够解放，机关效能还有待提高，投资环境还要进一步改善；等等。对这些问题，我们要高度重视，在今后的工作中采取有力措施，切实加以解决。

二、2008年工作任务

2008年是全面贯彻落实党的十七大精神至关重要的一年，也是我市实施“十一五”规划至关重要的一年。政府工作的总体要求是：以邓小平理论和“三个代表”重要思想为指导，全面贯彻党的十七大和中央、全区经济工作会议精神，紧紧围绕科学发展、加快发展、和谐发展，大力调整经济结构，转变经济发展方式，切实加强资源节约和环境保护，着力推进节能减排，继续解放思想，深化改革创新，努力扩大开放合作，更加注重改善民生，促进经济社会又好又快发展，推动富裕和谐新贺州建设，以优异成绩庆祝自治区成立50周年。

今年全市经济社会发展的主要目标建议为：在优化结构、提高效益、降低能耗、保护环境的基础上，生产总值增长12%左右；单位生产总值能耗降低2.2%，主要污染物排放总量减少（SO2削减950吨，COD削减5130吨）；财政收入增长12%左右；全社会固定资产投资增长23%以上；全社会消费品零售总额增长12%以上；外贸进出口总额增长10%以上；城镇居民人均可支配收入增长10%以上；农民人均纯收入增长9%以上；城镇新增就业人口1.2万人；人口自然增长率控制在8‰以内。

实现上述目标，不断缩小与全区平均水平、与周边发达地区的差距，我们必须坚持好字优先加快发展，着力推进经济发展方式转变；必须坚持以人为本加快发展，着力改善民生促进社会和谐；必须坚持改革开放加快发展，着力增强发展的活力和动力；必须坚持统筹协调加快发展，着力提高科学发展水平；必须坚持真抓实干加快发展，着力推进各项工作的落实。重点抓好以下八个方面的工作：

（一）坚持“工业立市”战略，强化工业对经济增长的主导作用

培育壮大工业支柱。突出抓好电子、有色金属开采加工、林产、建材、电力、食品药品加工等六大产业，大力开发上下游产品，延伸产业链，拓展配套产业，促进优势产业集群式发展。进一步“扶优扶强”，做强做大桂东电力，加快平桂飞碟公司、桂东电子、金源稀土功能材料公司培育上市步伐。加大工业项目实施力度，重点推进贺州火电厂、林浆纸板一体化等重大项目，确保桂华成公司钨矿复产以及吉光电子电解电容器、南华糖业等28项重点项目顺利推进和如期竣工。

加大工业技改和投资力度。鼓励支持企业运用高新技术和先进适用技术加快企业改造升级，积极推进珊瑚钨矿技改、梅林化工高目数重质碳酸钙超细粉、红星纸业特种纸技改二期工程、桂东电子铝箔技改、金源稀土技改工程等一批项目，力争全年完成工业技改投入增长25%以上。

加快发展园区经济。进一步完善“一廊两域三区一园”规划，完成电子科技工业园规划，力争新增2个自治区级A类或B类园区。多渠道筹措建设资金，完善园区基础设施，加快标准厂房建设。创新园区招商方式，重点引进产业带动优势明显、关联度高、辐射力大、配套协作功能强的龙头型大项目进入工业园区，加快形成产业集群。突出抓好旺高、信都和西湾（平桂）工业区的建设和管理，提高园区土地利用率和单位面积投资率、产出率，促进园区跨越式发展。

实施“中小企业成长工程”。培育扶持100家成长型企业，发展壮大30家强优企业、5家科技型企业，力争全市中小企业工业增加值增长22%。推进中小企业公共信息、融资担保、技术创新、社会信用、市场开拓、创业指导、人才培训等体系建设。

切实抓好节能减排。实施清洁生产，推广资源节约、替代和循环利用新技术，加快企业节能、降耗、减污技术改造，鼓励引导企业建设有利于节能、环保和资源综合利用的工业项目。以整治违法排污为重点，加大环保执法力度，加强对重点污染源、重点江河、饮水水源污染等的综合治理。深入实施“贺江清新”行动，抓好龟石水库水源地保护及水资源配套工程，确保完成自治区下达的年度约束性指标。

（二）加快城镇化步伐，着力塑造城市新形象

完善城市功能。进一步加强城市规划，依法按规划推进城市建设。按照“东西延伸，南北拓展”的城市建设思路，加快“一江两岸三新区”（即贺江及其两岸和江南新区、火车站新区、城东新区）建设，扩大城市规模。大力推进市区路网建设，加快推进八达西路延长线、灵峰南路、市区至姑婆山一级公路、火车站站前路至西湾（平桂）工业区一级路等城市主干道路建设，尽快实现对三个新区道路主骨架的全覆盖，增强城市辐射带动能力。加快市政项目建设，抓好新批建房地产项目的开工和在建的远东国际城、汇豪国际城、帝景湾小区、职教中心等项目的续建；启动旧飞机坪小区、江南经济适用房小区、火车站片区等一批项目的开发建设；进一步完善城市配套项目建设，抓紧推进管道燃气工程和城市污水综合处理厂、生活垃圾无害化处理厂二期工程、家畜定点屠宰场等重点市政项目。

加强城市经营和管理。完善城市长效管理机制，深入实施“城乡清洁工程”，继续抓好市容市貌、清洁卫生、环境污染、交通秩序、市场秩序、治安秩序等专项整治，加强城市管理综合执法，提高城市管理水平。完善城市建设投融资体制，做好土地收购储备和运营，推进城市基础设施和公共经营权的有偿转让，运用市场机制,吸引社会资本、外资参与城市建设。

加快县城和小城镇建设步伐。加快各县城路网、供电、供水、污水和垃圾处理等基础设施建设，强化县城对小城镇的辐射、服务与带动。加强城镇规划建设和管理，突出抓好信都、望高、黄姚、朝东等25个重点镇建设，构建市内大型区域性基础设施、高速路、铁路、高等级公路沿线的城镇密集带，吸引并培育发展各类生产要素，提高城镇化水平。

（三）大力发展现代农业，加快推进社会主义新农村建设

抓好农业灾后恢复生产工作。按照“点面兼顾、因地制宜、突出增收、落实技术、强化基础、优化服务”的要求，全力以赴抓好冰冻灾害后的重建和农业恢复生产。加快修复损毁的农业设施设备，保障农民安全饮水和灾后恢复农业生产用水用电。突出抓好种苗、化肥、农药、饲料等农资调运供应，满足农业灾后恢复生产需求。大力发展蔬菜、食用菌、家禽等短、平、快项目生产，增加灾区群众收入。加大科技支农力度，帮助农民解决生产自救和春季生产中的技术难题。做好春耕备耕服务工作，组织好春耕春种。抓好灾毁民房重建工作，确保在今年6月底前完成重建任务。

调整优化农业结构。在确保粮食安全的前提下，农业发展重点放到优化品种、提高质量上来，加快发展以品牌农业、创汇农业、生态农业、观光农业等为重点的特色农业，进一步扩大无公害蔬菜、脐橙、烟叶、马蹄、茶叶、梅李、松脂林、速丰林、桑蚕、食用菌等优势农产品基地。继续创建出口农产品检验检疫备案种植基地，加大农产品商标注册和组织名优农产品品牌申报力度，力争全年优势农产品种植面积扩大8-10万亩。加强林业生态建设，尽快恢复生态功能。大力推进农业机械化，提高农业生产效率。积极推广水产畜牧养殖规模化、标准化和品牌化经营，提高水产畜牧业在农业中的比重，促进农业增效、农民增收。

加快推进农业企业化。落实各项扶持政策，加大对农业龙头企业的培育和扶持力度，积极抓好农村社区股份合作、农村土地股份合作和农民专业合作试点，认真实施农业产业化“十百千万”行动，建立以“龙头企业＋专业合作组织＋专业大户”新型的产销关系和利益联结机制，力争全年新增规模农业企业30家、农民专业合作社5家。加强农产品贮藏、保鲜设施建设和农产品市场营销体系建设，促进农产品加工、销售，提高我市农业的市场应变能力。全面实施“科技兴农”战略，通过品种改良和新品种的开发、引进，以及农业先进实用技术推广普及和高新技术应用，全面提升农产品品质。大力推广农业标准化生产和生态农业技术，推进农产品原产地认证和质量安全体系建设，加快农产品质量认证步伐。

扎实推进新农村建设。继续抓好新农村建设试点工作，带动面上新农村建设。开展新农村建设指导员监督工作，重视发挥新农村建设指导员的作用。加强农村基层组织建设，通过开展“强乡带村”、“经济强村”和“三级联创”活动，巩固完善经济效益好、市场潜力大的产业，夯实新农村建设的经济基础。加大农民科技培训力度，不断提高农民的科技素质，全年完成培训农村劳动力10.5万人次，新增劳务输出3万人以上。加强农田水利、人畜饮水、农村电网、农村信息服务网络、乡村道路等农村基础设施建设，改善农村生产生活条件。推进龟石水库等病险水库除险加固工程，继续抓好龟石灌区节水改造和一批小型农田水利设施建设。加强土地管理，严格耕地保护。

大力发展县域经济。落实好发展县域经济的各项政策措施，着力培育“一镇一业、一村一品”的特色产业。统筹城乡产业布局，大力发展县域工业，加快县城工业集中区的建设。营造公平、良好的非公有制经济发展环境，加快发展县域个体、私营、民营等非公有制经济，使非公有制经济成为县域经济的主力军。

（四）巩固提高以商贸、旅游业为重点的第三产业，培育新的经济增长点

加快发展商贸物流业。加大市场培育力度，促进专业市场和大型专业批发市场的形成。积极打造加工贸易产业转移基地，力争全年新引进加工贸易项目30个以上。建立和完善商业网点审查审批规章制度，加强对商业网点的宏观调控，做好现代物流园区、6个大型专业市场规划并启动建设，推进工业园区周边住宿餐饮娱乐等综合配套设施建设。继续实施以龙头企业配送中心建设为重点的“万村千乡”市场工程，大力发展农村小卖部连锁店，开拓农村消费市场。

扩大对外贸易。加强对重点出口企业的指导和服务，不断提高出口商品的技术含量和市场竞争力。用好国家扶持政策，支持企业扩大出口，争取更多的中小企业、个体私营企业获得进出口经营权，更多地出口具有贺州特色的优势产品，培育新的出口增长点，力争全年进出口总额增长10%。

做强做大旅游业。以成功创建“中国优秀旅游城市”为契机，加大投入，进一步整合旅游资源，积极争取自治区批准实施桂台客家文化旅游贺州实验区，做好信都蕉树水库旅游项目前期工作，力争黄姚景区创国家4A级景区、客家围屋创国家3A级景区，继续完善秀水状元村和十八水景区基础设施，打造具有贺州特色的旅游精品。积极开拓国际、国内旅游市场，做好旅游、文化、商贸结合的文章，更好地发挥旅游带动作用。加强旅游行业管理和培训工作，提高服务水平，树立贺州旅游的良好形象。

培育发展现代服务业。积极发展邮电通信、家政服务、社区服务、中介服务等新型服务业，培植新的消费热点。鼓励民间资本广泛参与现代服务业发展，加快发展房地产业、金融保险、信息服务、文化产业等第三产业，完善提升服务业整体结构，满足人民群众的物质文化需要，扩大就业岗位。

（五）全力推进重点项目建设，拉动经济快速增长

加大项目实施力度。继续实施百项工程大会战，确保全社会固定资产投资完成164亿元，保持投资对经济增长的强劲拉动。突出实施好两轮六年、投资306亿元的交通基础设施建设大会战，加快打造以“东靠西连，南北贯通”大交通格局为新优势的桂粤湘三省（区）区域交通枢纽城市，全力推进桂梧高速公路、广贺高速公路、洛湛铁路、贵广快速铁路贺州段、昭平至蒙山黄村、八步经黄姚至昭平二级公路建设，开工建设贺州至姑婆山一级公路和一批通乡油路或水泥路，完善路网结构。加快市汽车客运中心、贺州港信都作业区建设。

推进重大项目前期工作。按照国家和自治区投资方向，重点上报和争取项目。着重推进和做好贵广快速铁路贺州段、永贺高速公路贺州段、贺州火电厂、贺州华润水泥厂、林浆纸板一体化、中石油3万立方米油库等重大项目前期工作，力争年内开工建设。继续抓紧风力发电场、稀土开发及深加工、肉食品深加工等项目前期工作。做好项目储备工作，筛选和储备一批关系贺州发展全局的重大项目，并做到常年筛选、动态跟踪、滚动储备，提高项目成功率。

多渠道筹集项目建设资金。尽快推进中小企业担保公司的组建，积极争取银行信贷支持，积极引导民间投资进入基础设施和社会事业项目建设。努力实施好贺州学院、贺州市中医医院利用外国政府贷款项目。加快市、县（区）职教中心、青少年活动中心、妇女活动中心、图书馆、博物馆等项目建设。

（六）加大招商引资力度，加快迎接东部产业转移基地建设

加强区域经济合作。要紧紧抓住中国——东盟自由贸易区加快构建、泛珠三角和泛北部湾经济区域合作加强、广西北部湾经济区发展规划获国家批准实施以及国际国内产业加快转移等多重机遇，以建设承接产业转移重要基地为目标，充分发挥贺州作为“三省通衢”的区位优势，坚持“走出去”、“请进来”相结合，主动融入粤港澳，拓展闽苏浙，挺进东南亚，全面加强与国内外在交通、能源、矿产开发、跨境旅游、农业等领域的合作，拓展合作关系，扩大合作规模，提升合作质量。

推进招商引资工作。强化招商引资目标责任制，建立健全招商引资绩效考评工作机制，创新招商引资工作方式，在抓好部门招商、专业队招商、挂职招商的基础上，继续推行产业链招商、以商招商等行之有效的办法，组建高素质、强有力的招商团队，加强与产业转出地的沟通，确保招商取得实效。完善部门分工协作机制和跟踪服务机制，做好重点招商项目的跟踪和督办。积极营造亲商、富商、安商的投资环境，确保项目引得进、留得住、有发展。认真落实“百企入桂”、“央企入桂”各项工作，突出抓好已签约项目的落实，协助做好项目报批、招投标、项目用地、征地拆迁等前期工作，确保项目如期履约，尽早开工建设。

主动迎接产业转移。紧紧抓住国家对东部和中西部加工贸易实行差别政策的有利时机，进一步完善我市迎接东部产业转移规划，找准产业转移的承接点，立足我市的比较优势推出一批优势产业招商项目，着力引进一批科技含量高、辐射带动性强、发展前景好的配套协作型龙头企业，推动迎接产业转移取得新的更大成果。积极向自治区争取在贺州及桂东地区开展东部产业转移基地基础设施建设大会战，特别是加快工业园区标准厂房建设，努力把工业园区打造成为我市接纳东部产业转移的主阵地。

（七）加强财税工作，努力保障重点支出

保持财政收入快速增长。积极培植财源，加大财政投入，通过贴息、担保、奖励、补助等方式，扶持特色经济、优势产业和强优企业发展，做强做大支柱财源；加强收入征管，严厉打击违法涉税行为，严格规范和执行国家税收减免政策，壮大各级财政实力；进一步完善财政收入增长激励机制，促进全市财政收入快速稳定增长。

努力保障重点支出。坚持按照市场经济和公共财政原则，优化财政支出结构，严格实行“收支两条线”管理。在保工资、保运转、保稳定的基础上，支持工业园区、重点产业和为民办实事项目建设；加大对“三农”和扶贫开发支持力度；支持国有企业技术改造、自主创新、节能减排和发展循环经济；继续加大对教育、社会保障、科技、文化、卫生、体育等公共事业的投入。

完善金融支持体系。进一步完善政府、银行和企业之间的联系机制，引导银行结合国家产业政策加大对我市重点项目、重点产业、社会发展薄弱环节的信贷投入。积极向上级争取金融机构升格，提高金融服务水平，发挥金融对地方经济发展的促进作用。积极吸引市外金融、保险、证券机构到贺州拓展业务，提高银行业、证券业、保险业的发展速度和质量。

（八）统筹推进以改善民生为重点的社会事业，促进社会和谐稳定

千方百计扩大就业。抓好城乡统筹就业工作，坚持以创业带动就业，支持自主创业、自谋职业和灵活就业。完善就业公共服务体系，改善就业环境，建成覆盖城乡的劳动力资源数据库。力争全市农村劳动力转移就业人数新增3万人，城镇就业人数新增1.2万人，城镇登记失业率控制在4%以内。强化就业指导和援助，重点做好下岗失业人员、零就业家庭、就业困难人员、失地农民、高校毕业生、退伍军人的就业安置工作。

完善社会保障体系。以基本养老、基本医疗、最低生活保障制度为重点，加快完善社会保障体系，落实机关、企业、事业单位基本养老保险制度，全面推进城镇职工、居民、低保和失业人员基本医疗保险、新型农村合作医疗制度改革，完善失业、工伤、生育保险制度，提高统筹层次。建立以财政供养为主的新型农村五保供养机制，全面推行“五保村规范管理活动”，切实加强乡镇敬老院基础设施建设和管理。扎实推进农村特困户危房改造工程和重点优抚对象危房改造工程。完善自然灾害应急救援和灾民生活救济制度、法律和就业援助制度。抓好廉租房、经济适用房建设和管理，年底前将廉租房制度覆盖到县城。

搞好扶贫开发。继续动员社会力量支持参与扶贫开发，大力推广参与式扶贫，凝聚扶贫开发的更大合力，扎实抓好第二批40个贫困村的整村推进扶贫开发，确保产业开发、改善生产生活条件和扶贫培训等任务的完成，力争今年解决1.5万人农村绝对贫困人口的温饱问题，农村低收入贫困人口脱贫2.4万人以上。加大库区移民工作力度，逐步改善库区移民的生产生活条件。

推进科技创新。全面启动第四轮科技创新计划，加快新产品、新技术的引进、开发和应用，建立健全创新体系，引导企业加大技术创新投入，增强企业核心竞争力和产品自主创新能力，加快高新技术产业发展。进一步加强知识产权保护工作。建立和完善科技创新奖励制度。加强科普工作，提高农村群众的科技教育水平。健全农村科技服务体系，继续抓好科技示范基地、示范乡镇、示范村和示范户的培育和建设。

坚持优先发展教育。加大对教育的投入，在继续改善农村中小学办学条件的同时，着力优化市区教育资源分布，进一步完善市区教育功能，大力推进义务教育均衡发展，保障贫困家庭学生、农民工子女、“留守儿童”平等接受义务教育。实施中等职业教育攻坚，加快驻梧中专学校整体搬迁贺州步伐。加快普通高中建设与发展。推进贺州学院建设，进一步提高高等教育质量。加强教师队伍建设。

实施人才强市战略。加强党政人才、企业经营管理人才、专业技术人才、社会工作者人才队伍建设。大力培养高层次、高技能人才和新型农村实用人才。创新人才引进、使用机制，优化人才发展环境。

加快发展卫生、文化、体育等各项社会事业。推进医疗卫生事业改革与发展，加强和完善疾病预防控制体系、职业病防治体系、医疗救治体系、卫生监督执法体系和农村卫生服务体系建设。以全面实施新型农村合作医疗制度为契机，完善各级新农合监管机制，加强新农合工作的监督指导。完善食品卫生安全应急体制，继续开展产品质量和食品安全专项整治，重点整顿药品药材市场秩序，保障人民群众饮食和用药安全。加快发展新闻出版事业，积极推进“农家书屋”建设，加强“扫黄打非”工作。扎实推进新时期“村村通”无线覆盖工程建设，不断提高农村广播电视覆盖水平。进一步完善公共文化服务体系，大力发展文化产业。深入开展全民健身活动，加强城乡基层体育设施建设，提高竞技体育水平。进一步做好老龄、妇女、儿童、残疾人、统计、人事、机构编制、工商管理、技术监督、税务、审计、人防、外事、侨务、对台、双拥、国防、物价、气象、防震减灾、应急、档案、修志、工会、住房公积金等各项工作。

进一步加强人口和计划生育工作。大力实施人口与计划生育“新农村新家庭计划”，深入开展“三无”创建活动，建立健全人口和计划生育工作新机制，不断完善利益导向政策，切实稳定低生育水平，提高出生人口素质，降低出生人口性别比。加大流动人口计划生育综合治理力度，争取在计生服务、依法行政和节育措施的落实上实现新突破。

推进“平安贺州”建设。继续开展“平安和谐贺州，铸造社会治安品牌”活动，严厉打击各种违法犯罪活动，增强人民群众的安全感。落实信访工作责任制和责任追究制，加强人民调解和法律援助工作，加大矛盾纠纷尤其是土地、水利和山林“三大纠纷”的调处工作，有效预防和妥善处置群体性事件。落实安全生产责任制，强化安全监管，严防重特大安全事故发生。完善应急管理体系，做好突发公共事件应急处置工作。整顿和规范市场秩序，强化市场监管，依法打击制假售假、虚假广告、商业欺诈、传销和变相传销、偷逃骗税等违法活动，净化市场环境。

加强民主法制和思想道德建设。依法做好村委换届工作，推行村务公开，推进城乡和谐社区建设，保证群众依法行使民主权利。深入开展“五五”普法，弘扬法治精神，形成自觉学法守法用法的社会氛围。深入推进社会主义荣辱观教育，广泛开展群众性精神文明创建活动，积极推进创建文明城市、卫生城市工作，不断提高全社会道德水准和文明水平。深入实施全民科学素质行动计划纲要，弘扬科学精神，普及科学知识。加强诚信贺州建设，不断提高全社会诚信水平。倡导城乡社会互助和志愿服务，努力形成社会和谐人人有责、和谐社会人人共享的生动局面。

三、努力建设服务型政府

以建设服务型政府为目标，以胡锦涛总书记视察广西重要讲话精神为动力，着力转变职能、理顺关系、优化结构、提高效能，形成权责一致、分工合理、决策科学、执行顺畅、运行高效、监督有力的行政管理体制。

（一）解放思想，加快转变政府职能。扎实开展继续解放思想大讨论活动，着力解决影响和妨碍科学发展、加快发展的思想观念和习惯做法，力求在思想观念上有新的转变，改革创新上有新的举措，科学发展上有新的成效，为民服务上有新的行动，班子建设上有新的面貌。健全政府职责体系，全面正确履行政府职能，努力建设服务型政府。在加强和改善经济调节、市场监管的同时，更加注重社会管理和公共服务，维护社会公正和社会秩序，促进基本公共服务均等化。

（二）科学决策，深入推行政务公开。完善公众参与、专家咨询和政府决策相结合的决策机制，对涉及经济社会发展的重大问题，事先要进行合法性、可行性论证，确保决策的科学性。建立健全社情民意反映机制、重大事项社会公示、听证、质询及新闻发布制度，加强市长热线、市长电子信箱、行风热线、政府网站等信息交流平台建设，不断提高政府工作的透明度和人民群众的参与度。

（三）从严治政，加强政府廉政建设。认真落实廉政建设责任制和领导干部述职述廉制度，强化对机关事业单位和国有企业主要负责人的经济责任审计，完善对权力的监督约束机制，注重从源头上预防和治理腐败。完善政府采购招投标中心，加强对政府投资的重大项目、政府采购以及国有资产并购等方面的监督，坚决杜绝暗箱操作、权钱交易行为。深入持久地开展反腐败斗争，切实提高拒腐防变能力，以清正廉洁的良好形象，赢得人民群众的信赖和支持。

（四）求真务实，提高政府工作效率。深化行政审批制度改革，加强各级政务中心建设。进一步加强公务员队伍建设，增强干部队伍政治鉴别能力、学习和思维能力、开拓创新能力以及执行能力。切实转变干部作风，抓好机关行政效能建设，推行行政问责制,落实首问负责制、限时办结制和责任追究制，努力提高政府工作质量和效率。进一步完善目标管理责任制，细化政府工作各项目标，逐级分解落实任务。加强对政府部门的规范化管理，完善对县（区、管理区）、部门目标管理责任考核办法和工作督查汇报制度，确保各项工作有计划、有部署、有落实、有成效。

各位代表，加快发展，任务艰巨；开创未来，重在当前。让我们高举中国特色社会主义伟大旗帜，以邓小平理论和“三个代表”重要思想为指导，全面贯彻科学发展观，落实党的十七大精神，在市委的坚强领导下，团结一致，振奋精神，抢抓机遇，开拓进取，努力实现经济和社会又好又快发展，为建设富裕和谐新贺州而努力奋斗！
